# Supplementary material for: Identification of photoperiod-induced specific miRNAs in the adrenal glands of Sunite sheep (Ovis aries)
Source: Front Vet Sci. 2022 Jul 22;9:888207. doi: 10.3389/fvets.2022.888207 (PMC9354845; doi:10.3389/fvets.2022.888207)
Supplement: Supplementary file 1 [file Data_Sheet_1.ZIP › Supplementary file/miRDeep2 file.zip!/miRDeep2 file/Know_Structure/LP42A3/oar-mir-323a.pdf]

miRBase precursor : oar-mir-323a
Total read count : 4347
oar-miR-323a-5p read count : 33
oar-miR-323a-3p read count : 4311
remaining reads : 3

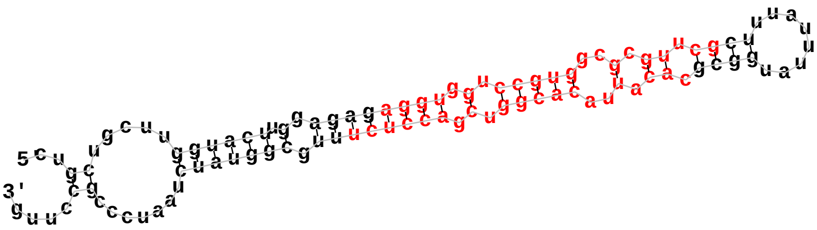

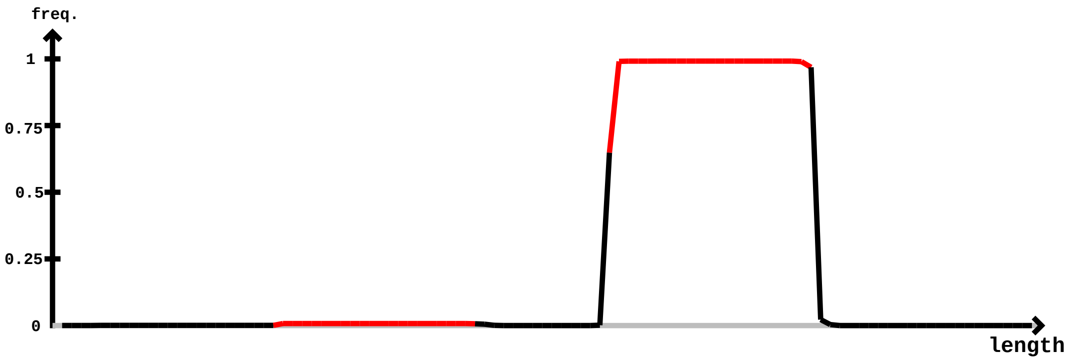


```
                     oar-miR-323a-5p
                                                  oar-miR-323a-3p
5'- cugcugcuugguacuuggagagaggugguccguggcgcguucgcuuuauuuauggcgcacauuacacggucgaccucuuugcgguaucuaaucccgccuug   -3'    exp
    .........((((((.(.((((((((.(.(((((..(.((.((((........)))).)).)..))))).).)))))))).))))))).............   reads   mm   sample
    cugcugcuugguacuuggagag...............................................................................   1       0    seq
    ...cugcuugguacuuggagag...............................................................................   2       0    seq
    ......................aggugguccguggcgcguuc...........................................................   4       0    seq
    ......................aggugguccguggcgcguucg..........................................................   7       0    seq
    ......................aggugguccguggcgcguucgc.........................................................   18      0    seq
    ......................aggugguccguggcgcguucgcu........................................................   4       0    seq
    .......................................................cgcacauuacacggucgaccucu.......................   6       0    seq
    ........................................................gcacauuacacggucgaccu.........................   4       0    seq
    ........................................................gcacauuacacggucgaccuc........................   56      0    seq
    ........................................................gcacauuacacggucgaccucu.......................   2708    0    seq
    ........................................................gcacauuacacggucgaccucuu......................   42      0    seq
    ........................................................gcacauuacacggucgaccucuuu.....................   3       0    seq
    .........................................................cacauuacacggucgaccu.........................   5       0    seq
    .........................................................cacauuacacggucgaccuc........................   36      0    seq
    .........................................................cacauuacacggucgaccucu.......................   1396    0    seq
    .........................................................cacauuacacggucgaccucuu......................   38      0    seq
    .........................................................cacauuacacggucgaccucuuu.....................   12      0    seq
    ..........................................................acauuacacggucgaccucu.......................   3       0    seq
    ..........................................................acauuacacggucgaccucuuu.....................   1       0    seq
    .............................................................uuacacggucgaccucuu......................   1       0    seq
```
